# *In Vivo* Quantitative Susceptibility Mapping (QSM) in Alzheimer's Disease

**DOI:** 10.1371/journal.pone.0081093

**Published:** 2013-11-21

**Authors:** Julio Acosta-Cabronero, Guy B. Williams, Arturo Cardenas-Blanco, Robert J. Arnold, Victoria Lupson, Peter J. Nestor

**Affiliations:** 1 German Center for Neurodegenerative Diseases (DZNE), Magdeburg, Germany; 2 Neurology Unit, Department of Clinical Neurosciences, University of Cambridge, Addenbrooke's Hospital, Cambridge, United Kingdom; 3 Wolfson Brain Imaging Centre, Department of Clinical Neurosciences, University of Cambridge, Addenbrooke's Hospital, Cambridge, United Kingdom; 4 Department of Psychiatry, University of Cambridge, Cambridge, United Kingdom; Penn State Hershey Medical Center, United States of America

## Abstract

**Background:**

This study explores the magnetostatic properties of the Alzheimer's disease brain using a recently proposed, magnetic resonance imaging, postprocessed contrast mechanism. Quantitative susceptibility mapping (QSM) has the potential to monitor *in vivo* iron levels by reconstructing magnetic susceptibility sources from field perturbations. However, with phase data acquired at a single head orientation, the technique relies on several theoretical approximations and requires fast-evolving regularisation strategies.

**Methods:**

In this context, the present study describes a complete methodological framework for magnetic susceptibility measurements with a review of its theoretical foundations.

**Findings and Significance:**

The regional and whole-brain cross-sectional comparisons between Alzheimer's disease subjects and matched controls indicate that there may be significant magnetic susceptibility differences for deep brain nuclei – particularly the putamen – as well as for posterior grey and white matter regions. The methodology and findings described suggest that the QSM method is ready for larger-scale clinical studies.

## Introduction

Iron – typically found in two oxidation states, Fe(II) and Fe(III) – is the most abundant nondiamagnetic agent in the human brain [1]. Iron is essential for normal brain function due to its role in oxidative metabolism and in activating a number of biological processes that enable formation and maintenance of neural networks as well as DNA synthesis and other enzymatic processes. In the ageing brain, however, relentless accumulation occurs as a result of iron misregulation [2], which in part, might be modulated by genetic factors [3]. Iron overload is thought to promote spontaneous release of highly neurotoxic free iron, which leads to the harmful formation of highly reactive radical species, thus drastically exacerbating oxidative stress [4], [5]. Such activity not only predisposes to neuronal death as part of the ageing process [6] but is also thought to be associated with neurodegenerative diseases [7]–[10]. It is unclear whether iron accumulation is the cause or a consequence of the neurodegenerative cascade, but it is widely accepted that monitoring the spatial distribution and the temporal dynamics of iron deposition may offer important insights for our understanding of neurodegenerative disease pathogenesis [11]–[13].

Studying brain iron in Alzheimer's disease (AD) is particularly relevant because iron overload is a well-known feature [14]–[18]; it is thought that in neurodegenerative diseases, iron homeostasis is seriously disrupted causing iron levels to increase [9]. Biological iron, however, is multi-faceted and as such, it might hold a number of roles in neurodegeneration. Iron is known to be a component of neuritic plaques [16], [19]–[21] and neurofibrillary tangles [22], and it has been suggested that an elevated iron milieu might constitute ideal proliferation and perpetuation environments for β-amyloid aggregation and neurotoxicity [23], [24]. The ability to accurately measure iron levels *in vivo*, particularly over multiple time points, therefore, may offer important mechanistic insights to help unravel the sequence of events that leads to neurodegeneration [11], [25]. If regional changes in iron concentration in the AD brain were found to be sufficiently robust, *in vivo* measurement could even offer a diagnostic tool.

Magnetic susceptibility is a fundamental electromagnetic property that has become relevant to the study of ageing and neurodegenerative disease because in the human brain, iron has been proposed to be the dominant source of contrast in quantitative susceptibility mapping (QSM) using magnetic resonance imaging (MRI) [26]–[28]. Given the newness of *in vivo* magnetic susceptibility measurements, particularly with respect to its possible application to neurodegenerative diseases, it seems germane to first review the physical background in some detail; this can be found as supporting information (Introduction S1).

Early MRI studies exploited a measure known as field-dependent transverse relaxation rate increase (FDRI) to assess the T2-related effects caused by magnetic susceptibility differences [29]. Promising results were shown in AD, where striatal substructures such as the caudate nucleus and the putamen were found to be sites of abnormal behaviour [17]. The calculation of FDRI, however, requires scanning at two different field strengths, severely limiting its clinical implementation. More recently, a single field strength strategy – known as calculation of susceptibility through multiple-orientation sampling (COSMOS) – was proposed [30]. COSMOS yields reliable magnetic susceptibility reconstructions but it is also cumbersome because it requires scanning at different head orientations. Previously, Haacke *et al.* had introduced susceptibility-weighted imaging (SWI), a postprocessing strategy that produces magnetic susceptibility weighted images by homodyne filtering and linearising T2*-weighted phase information prior to combination with magnitude data [31]. SWI is convenient, because it only requires the complex signal from a single gradient-recalled echo (GRE) acquisition, but lacks quantitative validity, because the resulting high-pass filtered phase is largely nonlocal and orientation dependent [32]–[34]. SWI has, nonetheless, already found a range of applications in the clinic such as enhanced visualisation of cerebral microbleeds, haemorrhages and other vascular alterations such as thrombosis as well as for detecting tumours, strokes or abnormal calcifications [35]–[37].

Presently, considerable effort is being made to develop and validate methods that can provide quantitative magnetic susceptibility information from the MR signal of single scan data [32], [38]–[43]. To this end, the present study aims to test for the first time a QSM framework to assess the spatial distribution of abnormal magnetostatic changes in Alzheimer's disease.

## Methods

### Ethics Statement

The study was approved by the Essex 1 Research Ethics Committee in the United Kingdom (Reference: 10/H0301/50).

Written informed consent was obtained from all the participants. Before inclusion, every patient was assessed by a neurologist (PJN). In the present context, although the target clinical population were patients suffering from a degenerative brain condition, we expected them to have capacity to consent as only the mild stages of Alzheimer's disease were studied. Note that we had ethical permission to scan patients who lacked capacity (with caregiver consent) but this was not needed in the present study.

### Subjects

Eight patients with early-stage probable AD according to criteria from the National Institute of Neurological and Communicative Disorders and Stroke and the Alzheimer's Disease and Related Disorders Association (NINCDS-ADRDA) [44] were recruited from the memory clinic at Addenbrooke's Hospital (Cambridge, UK). Eight matched controls and 3 young volunteers – one of which was scanned repeatedly on 3 sessions, each at least a month apart – also agreed to participate and were cognitively screened to exclude neurological or major psychiatric illness. Elderly controls performed normally on cognitive screening: mini-mental state examination or MMSE [45] and Addenbrooke's cognitive examination–revised or ACE-R [46]. The control exclusion criterion was ACE-R<88 (out of 100). All demographic details are summarised in Table 1.

**Table 1:** Group demographic profiles for all patients and healthy volunteers.

	Young controls (N = 3)	Elderly controls (N = 8)	Alzheimer's disease (N = 8)
**Gender, M∶F**	2∶1	5∶3	6∶2
**Age (years)**	32 (2)	70 (5)	72 (6)
**Education (years)**	N/A	14 (4)	15 (3)
**MMSE (/30)**	N/A	29 (1)	22 (4)
**ACE-R (/100)**	N/A	94 (4)	60 (14)

Demographic measures, where appropriate, are given as mean (standard deviation).

N/A denotes not applicable.

### Imaging

#### Equipment

All MRI experiments were performed using a 12-channel phased-array total imaging matrix (TIM) head-coil on a whole-body Siemens Trio 3T superconductive magnet with gradient coils capable of 45 mT/m and 200 T/m/s slew rate (Siemens Medical Systems, Erlangen, Germany).

#### Shimming

Mapping local field inductions in brain tissue can be selectively difficult due to the extreme field deviations originated at air/soft tissue/skull interfaces *e.g.* in orbito-frontal and anterior temporal areas. In the present protocol, ‘advanced shimming’ – an iterative field-mapping procedure implemented by the scanner manufacturer – was carried out prior to each SWI acquisition.

#### Susceptibility-weighted imaging

The SWI sequence used in the present study was analogous to that described elsewhere [47]. Fully-flow compensated, radio-frequency (RF)-spoiled (low flip angle/long echo time) 3D fast low-angle shot (FLASH) [48] volumes were acquired in true-axial orientation (*i.e.* slices perpendicular to the main field) using the following imaging parameters: repetition time (t_R_)/echo time (t_E_)/flip angle (α) = 35 ms/20 ms/17°, A matrix size of 256×240 with 1×1 mm^2^ in-plane resolution and 72 slices – 2-mm thick – achieved whole-brain coverage. Interleaving the slice-encoding order reduced crosstalk effects, while aliasing artifacts were minimised collecting 16 additional slices. Receiver bandwidth was set to 50 Hz/pixel and parallel imaging was enabled: the generalised, autocalibrating, partially-parallel acquisition approach (GRAPPA) [49] was used with an acceleration factor of 2 and 24 reference lines. The total scan time was 7 minutes. Each SWI complex dataset was combined using sensitivity maps derived from phased-array channel to body coil ratios [50].

Note that though t_R_ could have been shortened below 30 ms, we found experimentally that for α = 17° (and t_E_ = 20 ms) increased overall signal magnitude-to-noise ratio was attained with t_R_≥35 ms. Note also that while it was not the focus in the present study, the choice of doubling the slice thickness relative to the in-plane voxel resolution intended to optimally capture the venous system by ensuring that the magnetic field offset induced in the extra-venous space dominates the signal phase [31], [51], [52]. Aligning slices perpendicular to the main field also ensured certain sign coherence as the MR phase adds up more readily along the main field orientation; this is because the magnetic dipole fields have predominantly positive values along such direction.

#### Volumetric T1 imaging

T1-weighted anatomical images were also acquired in the same scanning session to resolve independently the underlying brain anatomy. The structural scan consisted of a 3D magnetisation-prepared, rapid gradient-echo (MPRAGE) [53] volume: t_R_/t_E_/inversion time/α = 2300 ms/2.86 ms/900 ms/9°, 192×192×144 matrix dimensions and 1.25×1.25×1.25 mm^3^ voxel size. Receiver bandwidth and echo spacing were set to 240 Hz/pixel and 6.7 ms, respectively. The total scan time was 7 minutes and 23 seconds.

#### Ultrafast T2 imaging

In addition, whole-brain, T2-weighted, half-Fourier acquisition, single-shot turbo spin echo (HASTE) [54] images were acquired to ensure that vascular pathology was not significant in any subject as for standard clinical routine. The following scan parameters were used: t_R_/t_E_/α/turbo factor  = 2000 ms/89 ms/150°/205; matrix, 320×256 (in-plane resolution: 0.7×0.9 mm^2^); 25 axial slices (thickness: 4 mm; gap: 0.8 mm); 5/8-phase partial Fourier transform; bandwidth and echo spacing of 401 Hz/pixel and 5.58 ms, respectively. GRAPPA acceleration was enabled with a factor of 2 after acquisition of 24 reference lines, resulting in a total scan time of 52 seconds.

For all scans, multi-channel RF coil sensitivity normalisation (*i.e.* prescan normalisation) was applied to compensate for inhomogeneous RF reception. In addition, in order to maximise acquisition consistency across subjects, the scanning bed was adjusted to co-localise the centre of the midsagittal thalamus with the scanner isocentre. The bed then remained stationary for the rest of the session. Furthermore, the field of view orientation for the MPRAGE and HASTE acquisitions was standardised for all subjects by alignment to stereotaxic space using the anterior and posterior commissures in the midsagittal scout image.

### QSM framework

A primer on QSM background theory briefly describing the derivation of the magnetic susceptibility dipole kernel and its related operator, , can be found as supporting information (Theory S1).

#### Background field extraction

Prior to inversion, the local self-demagnetising field induction, 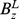, was inferred from the MRI signal. The distribution of magnetic flux densities can be expressed as an additive scalar field; thus: 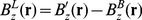
(1)where 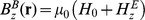 represents the background contribution to the observed induction field, and where 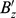 – the total magnetic induction experienced by nuclei – is proportional to the measured signal phase, 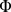: 
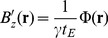
(2)
*γ* is the gyromagnetic ratio of the ^1^H nucleus and 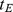 is the time to echo in a GRE experiment. Both bounded scalar fields, 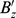 and 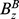, are characterised by field induction quantities that are orders of magnitude larger than those for 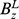; it has thus been proposed that 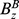 can be approximated by finding the distribution of magnetic dipoles – of freely varying strength originated outside the brain – that induces a magnetic-flux density distribution matching 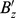 within the brain [38], [55], [56]. This is known as the effective dipole-fitting approach or projection onto dipole fields (PDF), and is in some way equivalent to extracting the harmonic contributions from the measured phase distribution [57], [58]. In matrix notation, where scalar fields bounded by the field of view are replaced by 3D real data arrays and scalar multiplications become Hadamard products, the problem can be stated as: 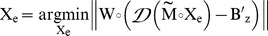
(3)



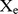
 is the magnetic susceptibility distribution that optimally satisfies the linear system in Eq. 3. The brain mask, 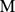, is a binary matrix that resolves brain tissue as a spatial distribution of unitary terms with zeroes elsewhere; hence the brain complement mask, 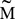, obtained by subtraction from the identity matrix, 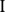, *i.e.* 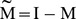, isolates the exobrain space. A pointwise multiplication with 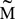, therefore, followed by a dipole kernel operation, , denotes that the minimiser tries to solve for a magnetic dipole distribution in the extra-cerebral space that matches the observed internal (brain) induction field. A brain-masked weighting matrix, **W**, is also needed to incorporate brain-only information to the solution and to compensate for noise variance spatial nonuniformities in the signal phase [59]. As previously demonstrated, **W** can be derived from the inverse of the standard deviation of the measured phase, which is, in turn, proportional to the signal magnitude [38], [39], [60].

**Figure 1:**
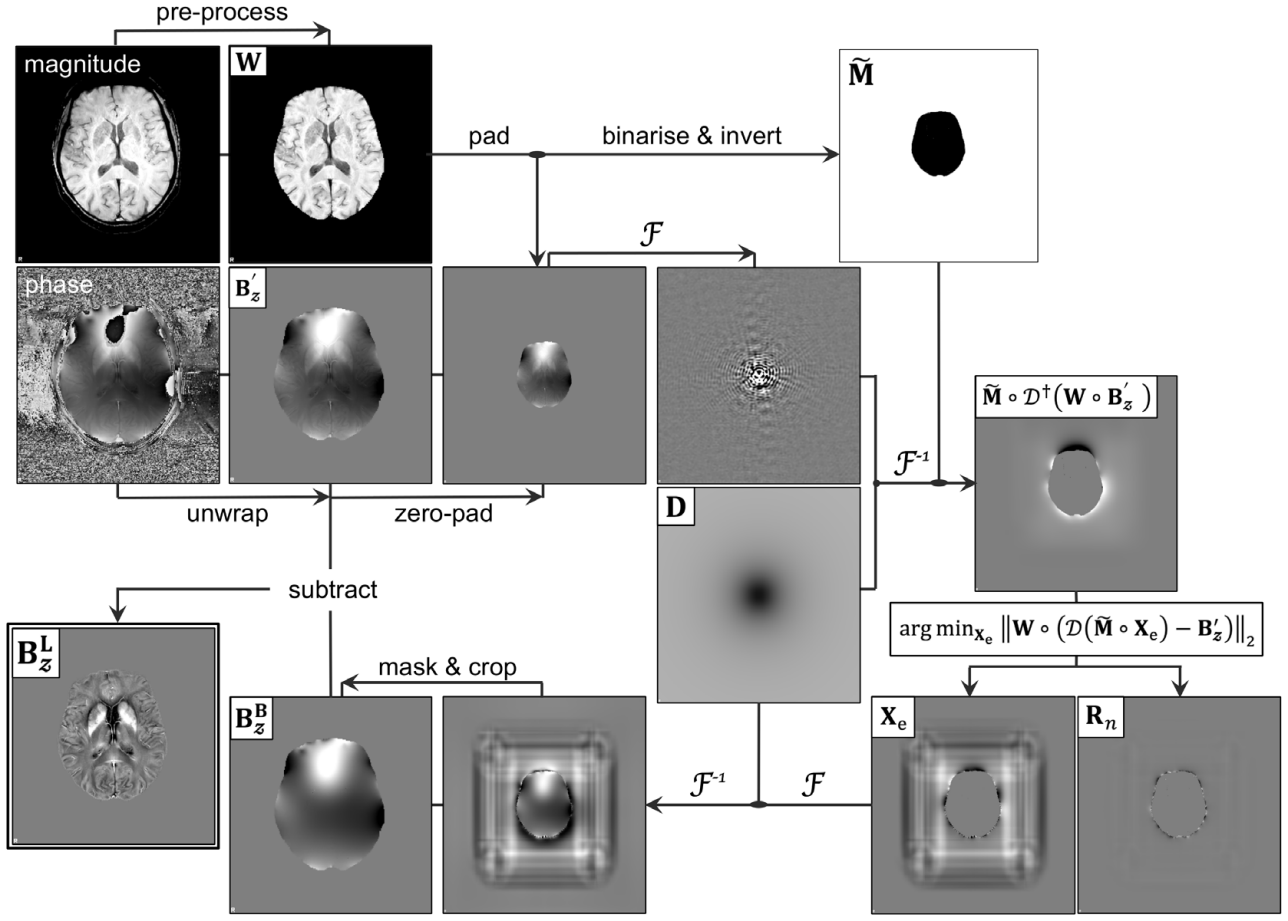
QSM pre-processing. Schematic depiction of the projection onto dipole fields (PDF) pipeline implementation. **W**: weighting matrix; 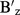: (measured) magnetic induction; 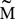: exobrain mask; 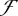: Fourier operator; **D**: magnetic dipole kernel; 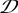: dipole operator; 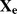: (estimated) exobrain dipole distribution; 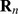: (minimised) conjugate gradient residual matrix; 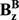: (estimated) background field; 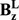: (estimated) foreground field.

The shimming residual was estimated using a method based on the PDF principle [56], depicted schematically in Figure 1. The conjugate gradient algorithm [61] is highly efficient at solving large-scale linear systems, hence it was used here. Each conjugate gradient iteration is a successive approximation to the solution, where residuals are updated (as 3D arrays) along a previously inferred search direction, and search directions are updated (also as 3D arrays) with the newly computed residual. The algorithm was terminated when the Euclidean norm of the conjugate gradient residual matrix for the *n* ^th^ iteration, 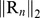, satisfied the following criterion: 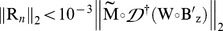
(4)where the right-hand side term is proportional to the norm of the starting residual, *i.e.* a thousandth of the overall field perturbation outside the brain inferred from the inner-brain measured induction field. Note that the dagger in superscript denotes the Hermitian transpose operator.

The optimal background induction field along the *Z*-axis – defined in matrix form as 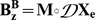 – was then introduced in a matrix-equivalent expression of Eq. 1 to infer 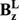.

For comparison, SWI-style high-pass filtering [31] was also carried out. Seven different isotropic Hanning window sizes were tested: 32-, 64- and 128-voxels-wide in 2D and 3D, and 100×100 for direct comparison with the standard SWI filter width implemented in the scanner console.

#### Brain mask calculation

Brain masks were derived from the magnitude image using the brain extraction tool (BET v2.1) [62] with fractional threshold set to 0.2. Brain masks were further eroded by convolution with a 6×6×6-voxels-wide cubic kernel using fslmaths (BET2 and fslmaths available from the FMRIB's software library [63], FSL v5.0.1 – http://www.fmrib. ox.ac.uk/fsl).

#### Weighting matrix

The noise weighting matrix, **W**, was derived from the brain-extracted magnitude image, which was then RF-bias field inhomogeneity corrected using N4-ITK [64] (available from the advanced normalisation tools software package, ANTs v1.9.x – svn release 793++, http://www.picsl.upenn.edu/ANTS) with default arguments. The preprocessed magnitude image was finally normalised to a control posterior ventricular region free of partial volume contamination whereby the mean weighting factor was downscaled to 1 by a double-precision division. Details on reference region delineation can be found below.

#### Phase unwrapping

MRI phase data is wrapped around 2π; thus, in order to apply the PDF approach, signal phases were first unwrapped. We implemented a direct method that simply applies the Laplace operator to take advantage of the phase's trigonometric properties [65]; this leads to: 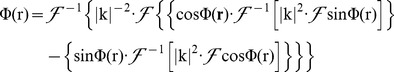
(5)


As previously noted, nulling the phase's Laplacian not only spatially unwraps the phase distribution, but also partially filters out components from the background field [66], [67]; which, to some extent, is also expected to satisfy Laplace's equation [57], [68]. The Laplacian-based method has been shown to be preferable for QSM than a recently proposed 3D best-path unwrapping algorithm [69], with the added advantage that the computational overhead is reduced to performing only eight fast Fourier transforms.

#### Regularised inversion strategies

The truncated k-space division method has been proposed to overcome some of the issues that arise from direct field induction-to-susceptibility inversions [32]; though with data acquired at a single head orientation, Bayesian regularisation approaches are superior, yielding susceptibility maps that are highly comparable with those reconstructed from multiple head-orientation experiments [40]. Specifically, those that incorporate spatial priors derived from the magnitude image have become popular due to their improved ability to attenuate noise amplification and streaking artefacts [38]–[43]. The objective function in such minimisation schemes typically consists of two terms: (i) a data fidelity constraint with noise nonuniformity correction and (ii) a regulariser, which enables the solution to share sharp edges with the magnitude image while promoting susceptibility compartmentalisation elsewhere.


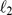
-*norm regularization*. The quadratic 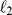-norm (or Tikhonov-like) minimisation approach is a well described conditioning framework for the field-to-source inversion problem, and was first proposed in this context by Kressler *et al.* [39] and de Rochefort *et al.* [38]. In this study, we improved the conditioning of the system, as recently suggested, with the application of a 3D gradient operation to the solution [42]. In Lagrangian form: 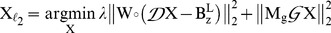
(6)


where the action of a linear finite-differencing (or total-variation) operation, 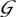, acting in the three directions of space, is masked by 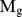 – a binary matrix that excludes sharp edges in the 3D gradient transform of the magnitude image. In other words, the regularisation term ignores susceptibility boundaries where the magnetic dipole kernel approximation is likely to break down [70]. *λ* is the associated regularisation parameter that trades data fidelity with solution smoothness.


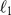
-*norm regularisatio*. It is known that 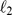-norm regularisers promote a large number of small spatial gradients to the solution [71]. But it can be realistically assumed that magnetic susceptibility compartments in the human brain vary smoothly across regions with homogeneous chemical composition; hence a regularisation strategy that promotes “sparsity” in the gradient transform of the source distribution appears to be preferable in this context. Note that the term “sparse” here refers to a large number of zero-gradient components. In analogous scenarios, in order to address this issue, 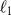-norm regularisers have been proposed to penalise small values in favour of fewer large coefficients [71]–[75]. The 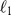-norm conditioning to the magnetic susceptibility inversion problem – known as morphology-enabled dipole inversion (MEDI) [41], [42], [76] –, which also incorporates physical priors derived from the magnitude image, can be formulated as follows: 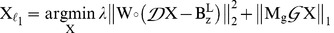
(7)


#### Iterative minimisation for dipole inversion

In order to apply efficient numerical methods, the cost functions of the constrained convex problems stated in Eq. 6 and 7 can be expressed as unconstrained minimisers by expanding the 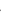
- and 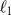-norm definitions and deriving by the solution [42]. Note that the sparsifying binary mask, 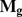 – used to supress voxels where signal magnitude gradients are exceedingly large – was inferred by applying a threshold to the sum of gradients whereby 30% of brain tissue was labelled as edge [42].

The unconstrained linear problems were solved using the conjugate gradient algorithm to compute the descent direction and a backtracking line-search strategy [77] to cheaply approximate the minimiser at every iteration; this is analogous to the method employed by Lustig *et al.* [71] in the context of compressed sensing, and should be equivalent to Newton-step based strategies proposed elsewhere [39], [42].

A range of regularisation parameters were used with the 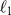-norm inversion scheme: *λ* = 200 (attenuated solution due to heavy regularisation), 300, 500, 750, 1250, 1750, 2250, 2750, 3250 and 10^4^ (where the effect of the regularisation term was fully suppressed resulting in severe streaking artefacts). In this study, a range of parameters was deemed optimal based on the assumptions that: (i) the residual of the consistency term must be minimal [39]; (ii) reconstructions should yield little overall solution attenuation [38], [39], [78]; and should also lead to (iii) consistent group differences as well as (iv) a stable young control group range. For direct comparison, an 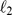-norm regularisation parameter was chosen whereby the mean susceptibility value in the globus pallidus for the elderly control group matched that from an 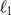-norm reconstruction within the optimal parameter range.

Regularisations were stopped when the absolute residual difference between two successive iterations reached: 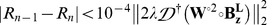
(8)


where the squared-norm represents the least-square residual after the first iteration.

#### Manual delineation of reference regions

It should be noted that inferring magnetic susceptibility distributions using (approximated) dipole kernel operations does not yield absolute bulk magnetic susceptibilities; this is due to the singularity at the origin of the k-space kernel (Theory S1), which translates to unknown induction field offsets for each reconstruction [70]. Hereafter, therefore, 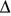 will be used to denote “relative (to a reference region)” measurements calculated using underdetermined dipole kernel operations.

**Figure 2:**
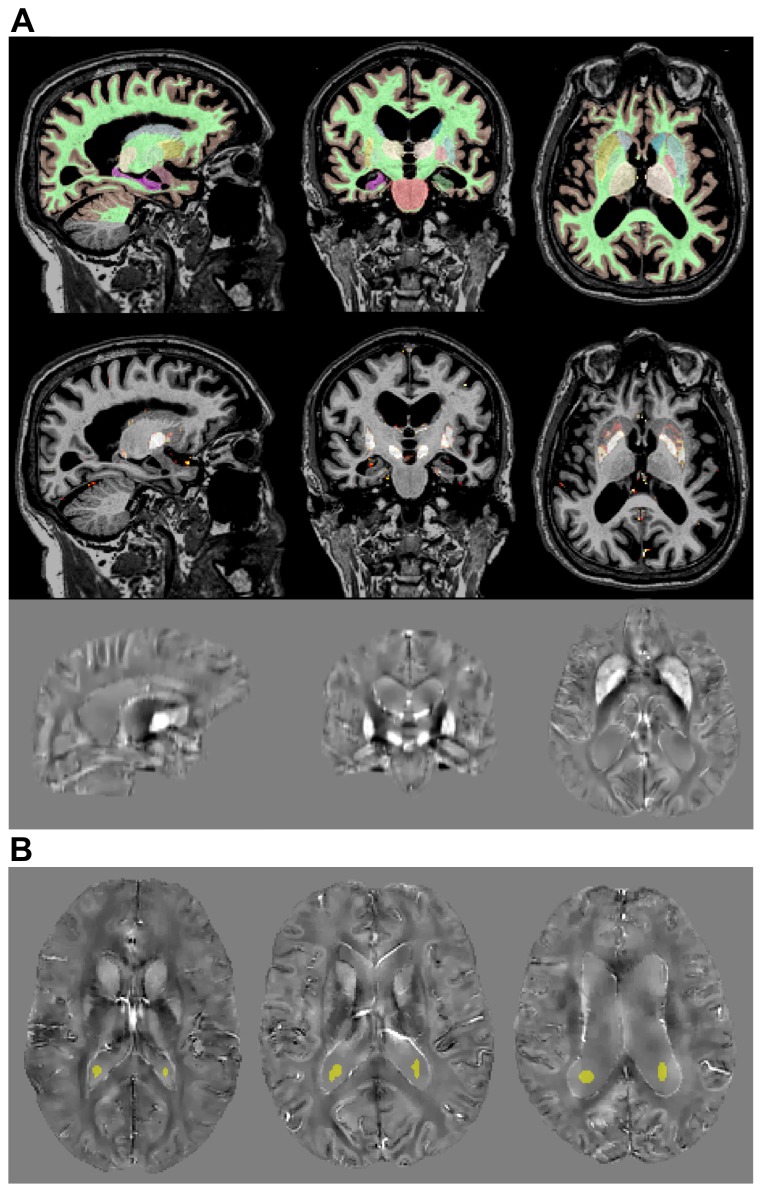
Regional QSM data extraction and reference selection. (A) FIRST's automated regional data extraction from magnetic susceptibility maps after alignment to structural space. (B) QSM reference region (yellow) for a young subject (left), an elderly control (middle) and an AD patient (right). Note the avoidance of large QSM spatial gradients and the slight mask size adjustment according to ventricular size.

For accurate and comparable QSM measurements, therefore, a reliable reference region must defined. In this study, we chose to baseline at 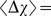 0 ppm the magnetic susceptibility of a homogeneous bilateral posterior ventricular region elongated along the *Z*-axis; such region was manually delineated using MRIcron v12/2012 [79] directly on reconstructed QSM images, and selectively excluded heterogeneous behaviour as well as partial volume contamination from ventricular boundaries and calcifications. Besides, for patients presenting with ventricular enlargement, the reference region was expanded whereby the minimum distance between the edge of the reference mask and the boundary of the ventricular space was within [3]–[10] voxels (Fig. 2B).

#### Workstation and development software specifications

All data postprocessing was performed in a laptop computer with a 2.6 GHz quad-core CPU, 8 GB DDR3 RAM and a solid-state drive running OS X v10.8 (Apple Inc., Cupertino, CA, USA). Processing algorithms and statistical analysis – except where stated otherwise – were prototyped in-house using Matlab R2012a (The Mathworks Inc., Natick, MA, USA).

### Structural MRI data processing

#### Spatial coregistration and tissue segmentation

In order to enable regional extraction using T1-weighted anatomical information and to perform whole-brain analysis of QSM data, rigid registration and nonlinear warping to a standard space were performed using SPM8 (http://fil.ion.ac.uk/spm). Prior to this, datasets were preprocessed as follows: first, the origin for each dataset's coordinate system was set to the anterior commissure. Then, MPRAGE volumes were skull-stripped using the hybrid watershed algorithm (HWA) with atlas information [80] (available from FreeSurfer v5.1.0, http://surfer.nmr.mgh.harvard.edu); this was followed by RF-bias correction using N4-ITK [64], which performs best in the absence of spurious signals from skin, fat and venous sinuses. Note that RF-bias inhomogeneity correction is known to improve spatial normalisation in the context of voxel-based morphometry [81]. In this study, however, N4-corrected signal intensities were substituted into the original magnitude image to reconstitute a bias-corrected whole-head dataset, as it was found experimentally that the inclusion of cranial information improved the performance of rigid coregistrations. RF-bias corrected T2*-weighted magnitude information was also inserted into the original image for coregistration. In addition, it was found experimentally that the “clean-up partition” feature in SPM8's unified segmentation (“Segment”) implementation was successful at eliminating spurious mis-segmentation of venous sinuses into grey matter partitions, resulting in visually improved tissue maps and spatial warping; this option was therefore enabled in the present study.

Grey and white matter tissue probability segments as well as bias-corrected (N4-ITK+SPM8) whole-head structural images were stored in native MPRAGE space. The former were used for whole-brain tissue extraction; whereas the latter, which showed improved signal intensity homogeneity for extra-cerebral tissue, were used for rigid alignment with N4-corrected whole-head T2*-magnitude images.

With the aforementioned exceptions, SPM8 coregistrations were performed using default settings. Subsequently, magnetic susceptibility maps were aligned to anatomical space using the resulting rigid transformation field, and were warped into MNI152 space (Montreal Neurological Institute, McGill University, Canada) applying the composition of rigid and nonlinear fields. Both spatial transformations were followed by interpolation using a trilinear operator.

#### Automated regional extraction

Deep grey matter structures were segmented automatically from skull-stripped (HWA) and RF-bias corrected (N4-ITK) MPRAGE volumes using FSL-FIRST [82] – a Bayesian statistical model that incorporates prior knowledge to constrain a set of deformable surfaces. QSM values were extracted bilaterally from thalamic (Thal), caudate nucleus (Caud), putamenal (Puta), globus pallidus (Pall), hippocampal (Hipp) and amygdalar (Amyg) regions as computed by FIRST; and from whole grey- and white-matter (GM/WM) masks inferred from SPM8 tissue segments that were binarised (thresholding at Prob = 0.5), intersected with the aligned T2*-derived brain mask, and excluded subcortical voxels that had previously been assigned by FIRST. On visual inspection, FIRST performance was robust in the presence of severe atrophy (Fig. 2A). Note that all regions of interest were deemed appropriate by a neurologist (PJN); though slight underperformance was observed in the delineation of the globus pallidus. This is due to poor T1-weighted contrast, but it should be highlighted that the prior knowledge information built into FIRST yielded reasonable estimates.

### Statistical analysis

#### Regional study

After whole-brain QSM normalisation to a reference value and rigid coregistration to structural space, regional information was extracted for each subject and was plotted as (normalised) group histograms for both regularisation types (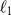
- and 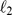-norm). Some distributions were skewed, hence median magnetic susceptibility values for each AD patient and age-matched control were used for statistical testing. Data for each regularisation parameter and for each region of interest were compared using nonparametric Wilcoxon rank-sum–*i.e.* Mann–Whitney *U*–tests [83]. As there was no a priori assumption about the directionality of QSM changes, tests were computed two-tailed. Rank-sum values that survived a lenient statistical threshold of P = 0.05 were plotted in the results, but a more stringent threshold of P = 0.005 was used to partially correct for multiple comparisons (equivalent to Bonferroni-P = 0.05 on n = 10 tests).

Information from whole-brain data consistency residuals and from the regional analyses were assessed together to identify an optimal 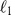-norm regularisation parameter range. In order to further assess differences across parameterised solutions, QSM values from a highly abnormal region were plotted for each subject as a function of the fidelity-regularisation trading weight, *λ*. Group averages and standard deviations were also plotted to estimate QSM attenuation due to excessive regularisation in relation to cross-sectional and serial data dispersion. Furthermore, median QSM subject values for a selected region were plotted against a major hallmark of AD, hippocampal atrophy – a measure of neuronal loss [84]. FIRST-segmented hippocampi, after satisfying a further visual inspection, were normalised by total intracranial volumes [85] determined using a previously validated method [86].

#### Whole-brain study

A selected 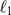-norm reconstruction was tested for whole-brain analysis. Prior to performing statistics, in order to reduce the effect of coregistration errors in spatial normalisation, QSM reconstructions were smoothed by convolution with an 8-mm full-width-at-half-maximum, isotropic (3D) Gaussian kernel. Finally, permutation-based FSL-randomise v2.9 [87] with threshold-free cluster enhancement or TFCE [88] was used to perform nonparametric, cluster-based, cross-sectional comparisons between AD patients and elderly controls. Running 12,870 permutations of the data achieved exhaustive testing, and results were shown at a statistical threshold (corrected for multiple comparisons) of P_TFCE_ = 0.05.

## Results

### Background field removal

**Figure 3:**
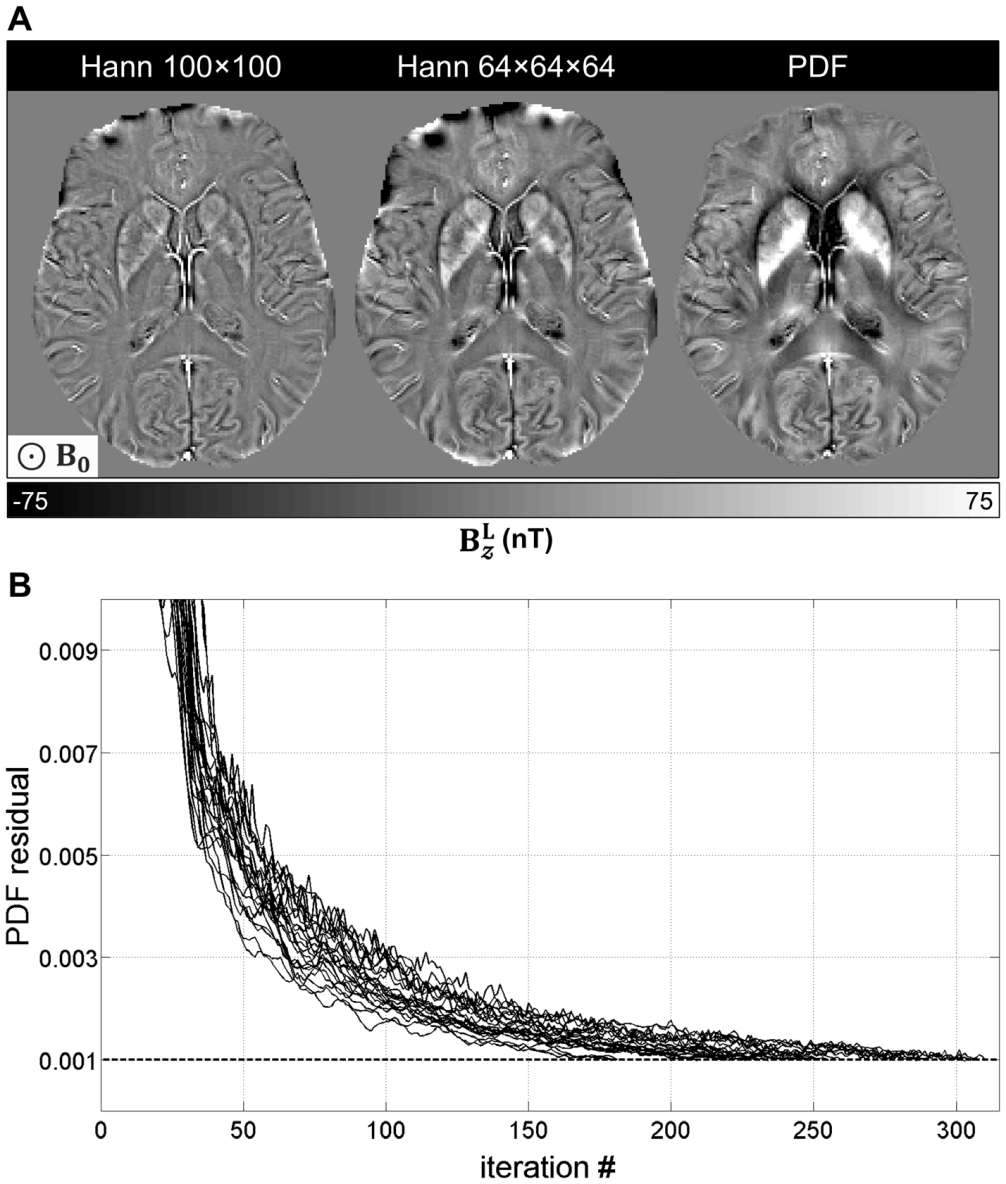
Differential performance of background field extraction methods. (A) Low-frequency field removal using Hanning and PDF filtering. The effective dipole-fitting approach reduces edge artefacts while largely preserving local perturbations elsewhere. (B) PDF's conjugate gradient convergence pattern. All N = 21 experiments similarly approached the proposed tolerance limit.

The PDF approach was qualitatively compared with homodyne filtering. Figure 3A illustrates that PDF-derived local induction maps are superior to those inferred from SWI-style filtering; the latter showed edge artefacts and attenuated contrast throughout. All other Hanning window sizes tested here also led to undesirable results.

In order to solve the PDF problem stated in Eq. 3, the unsupervised conjugate gradient algorithm searched iteratively for an optimal solution until a minimum normalised residual value was achieved (Eq. 4). Fitting residuals were plotted (Fig. 3B) to illustrate their robust convergence; all minimisations were terminated within [180–310] iterations. The average processing time to reach such tolerance was 19 minutes.

### QSM data consistency and test-retest reproducibility

#### Data fidelity optimization

**Figure 4:**
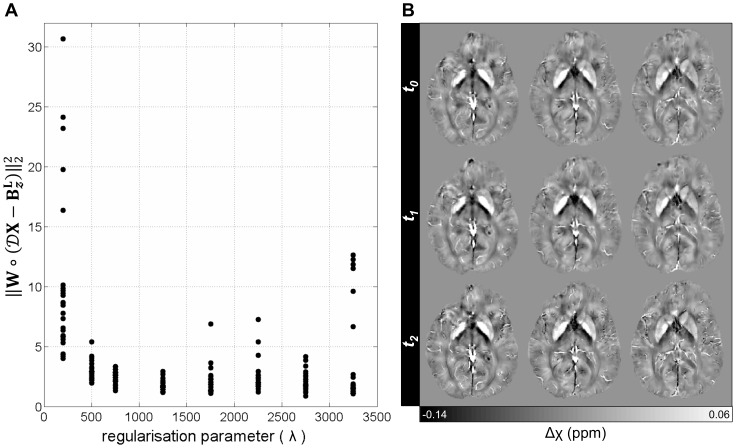
QSM data fidelity and serial measurement robustness. (A) Data consistency modulated by the choice of regularisation parameter. Data points represent data fidelity residuals for each reconstruction. *λ* = 10^4^ (not shown) led to the most incoherent solutions. The overall concave shape of the data, with a well-defined global minimum, points at optimally constrained reconstructions with *λ*≈1250. (B) Rigidly realigned magnetic susceptibility maps (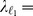
1250) for a young control scanned in three sessions (time-points: *t_0-2_*). Serial behaviour on a single subject was deemed highly robust.

The data consistency term in regularised inversion schemes constrains the problem to finding optimal solutions that are faithful to the spatial distribution of local field inductions. The squared 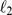-norm of such term was plotted (Fig. 4A) for several 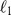-norm regularised solutions. Heavily regularised (*λ*<500) or under-regularised (*λ*>3250) solutions were the most unfaithful to the measured data, whereas the highest fidelities were observed for *λ* = 1250 and 750, respectively. The average processing time for 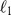-norm reconstructions at the proposed residual tolerance (Eq. 8) was 7.5 minutes.

#### Serial stability of MEDI's 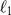-norm QSM inversion

A young volunteer was scanned on three occasions to assess the test-retest reliability of QSM reconstructions. On visual examination, the maps produced across multiple sessions were highly comparable (Fig. 4B).

### Qualitative assessment of 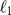
- and 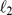-norm regularised inversions

**Figure 5:**
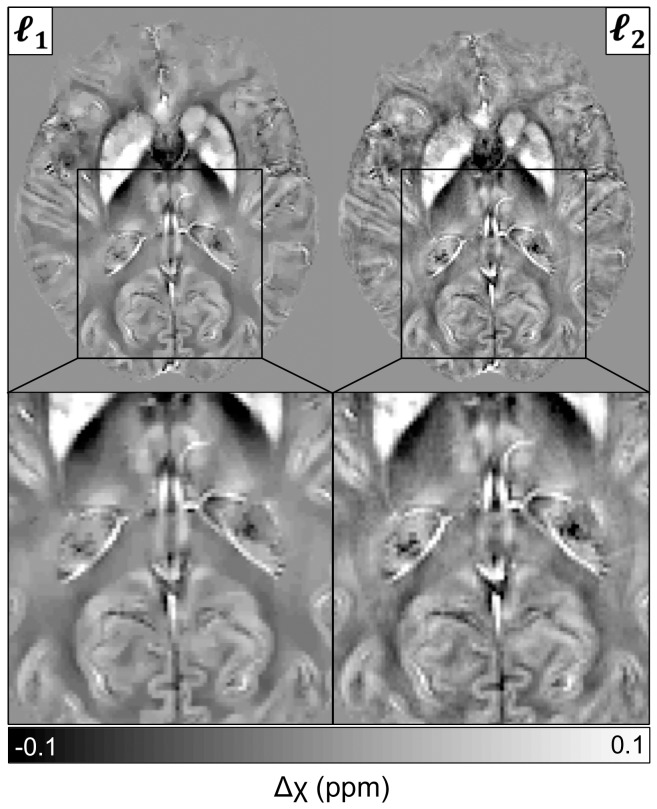
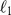
- versus 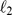-norm QSM reconstructions. The 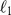-norm approach yielded better-compartmentalised maps. The 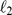-norm method preserved more anatomical detail.

Susceptibility maps were also reconstructed using the previously described 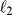-norm regularisation strategy (Eq. 6). 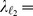
300 yielded median values in the globus pallidus that matched those for 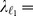
1250. Regularisation using the 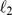-norm of the solution's gradient transform usually requires fewer conjugate gradient runs to reach the stopping criterion, which leads to faster convergence (2.5 minutes on average for the present dataset); but it also promotes smaller gradients to the solution, which leads to noisier reconstructions. As can be noted in Figure 5, gradient sparsity enhancement by 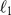-norm regularisation results in a slightly more compartmentalised magnetic susceptibility distribution (*i.e.* smoother behaviour between sharp susceptibility interfaces), which is preferable in theory, but it might be inferior to resolve subtle anatomical detailing.

### QSM in Alzheimer's disease

#### Regional study

**Figure 6:**
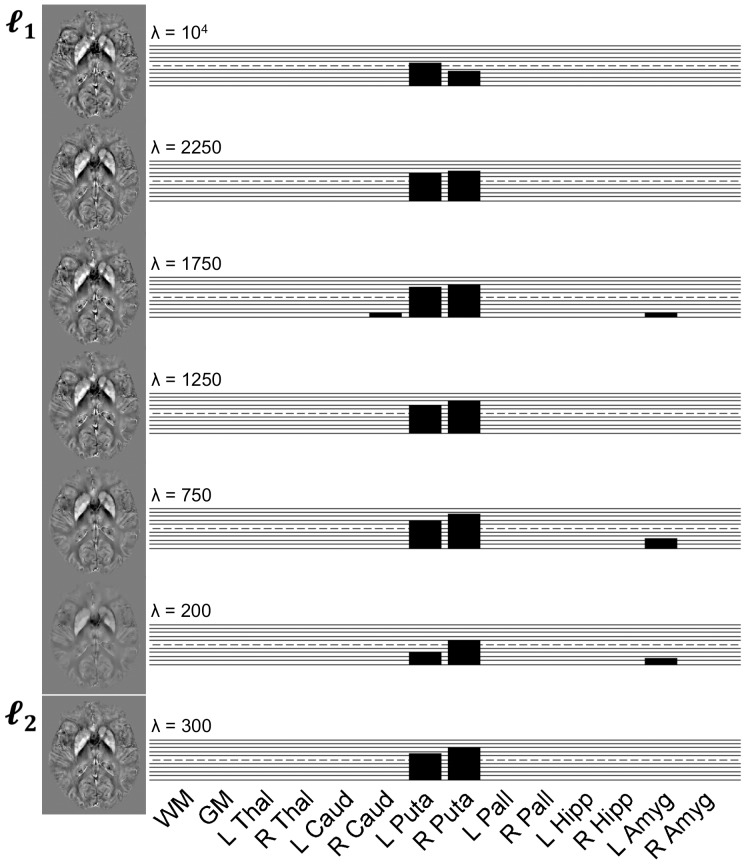
Regional QSM group results. Permutation-based statistical comparisons between AD and control groups in eight regions of interest for six 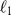
- and one 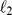-norm regularisation schemes. Each bar represents an absolute sum-of-ranks difference relative to that for P = 0.05 (if surviving such threshold); each solid horizontal line represents +2 (sum of ranks); and the discontinuous line marks the sum of ranks returning P = 0.005.

Median magnetic susceptibility values inferred from AD patients were compared with those from matched controls using nonparametric statistics. The cross-sectional results are summarised in Figure 6 for several inversions, from which it was confirmed that both putamena (right slightly worse than left) are highly abnormal in early-stage AD (P<0.005 for most comparisons). The left amygdala and right caudate also showed increased magnetic susceptibilities, though the effects were less pronounced (P<0.05). It was noted that regularisation parameters in the 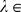 [750, 2250] range yielded the strongest differences. In addition, matching 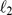
–norm data also yielded strong bilateral magnetic susceptibility differences in the putamen.

**Figure 7:**
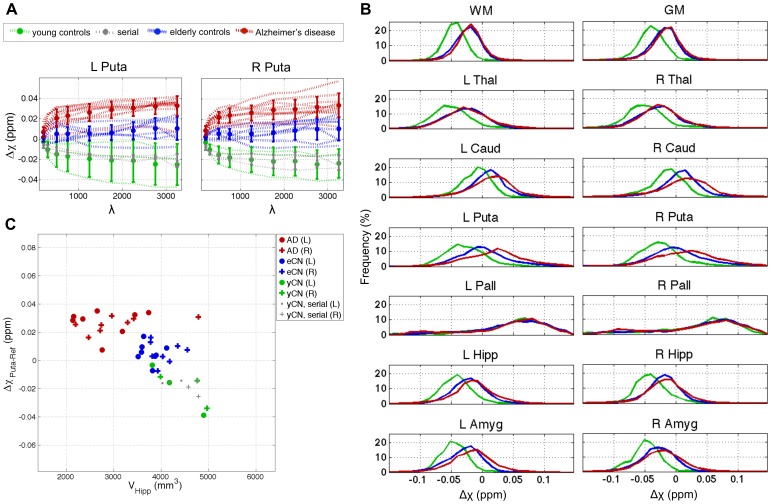
In-depth regional QSM data assessment. (A) Median susceptibility values from the bilateral putamen for the AD and control groups plotted as a function of regularisation parameter, *λ*. Discontinuous lines represent linearly interconnected data points for each subject, whereas solid dots and error bars describe group means and standard deviations. 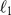-norm regularised 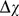 values are stable across a large range of parameters; though strong dependency was found for *λ*<750. Upward trends from young to adult and from healthy to AD, almost complete separation between patients and controls, and narrow serial measurement dispersion were also clearly visible. (B) Histogram plots for regional data from 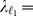
1250. (C) Median putamenal magnetic susceptibility values plotted against hippocampal volumes for all subjects in the study.

The strong regularising penalty imposed by small *λ*'s results in heavily attenuated magnetic susceptibilities [38], [39], [78]. This was also observed in this study (Fig. 7A), where median putamenal values for each subject and overall group means were plotted against *λ*. The data suggest that *λ* = 750 is the QSM attenuation “tilting point” for the present implementation, which is concordant with the last result and with Figure 4A. The plots also led to three additional observations: (i) the AD patient data range in the putamen barely overlapped with that from all other control subjects; (ii) young control values were lower than the lower-tail of elderly control data; and (iii) serial data points (centred in the young control range) were less dispersed than those from any other group.

For simplicity, at this stage, 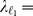
1250 was deemed optimal; hence its derived histogram plots for each group and region of interest were selectively shown in Figure 7B. Qualitative examination confirmed the effect (increased magnetic susceptibility) in both putamena, not only as function of disease but also as a function of age. Similar trends – *i.e.* susceptibility distributions in patients more dispersed towards higher values than those from controls – were also observed (in order of prominence) in the caudate nucleus, amygdala and hippocampus. In addition to these, qualitative differences between the two healthy groups were also apparent in the bilateral thalamus, and in whole grey and white matter. Remarkably similar behaviours were also observed for parameter-matched 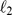-norm data (data not shown).

To complete the regional exploration, putamenal susceptibility values were plotted against FIRST-derived hippocampal volumes in Figure 7C. Hippocampal atrophy – a well-known AD feature – was significant (P<0.005) for the AD cohort studied here relative to matched control data. The distribution of individual volumes showed little – but some – overlap between patients and healthy controls. The scatter plot suggests that in AD, hippocampal atrophy does not predict magnetic susceptibility deviations in the putamen. Some subjects showed atrophy but little magnetic susceptibility change, whereas some other patients presented with relatively preserved hippocampi and strong susceptibility alterations. The combined data, however, resulted in complete separation between groups.

#### Cluster-based analysis

**Figure 8:**
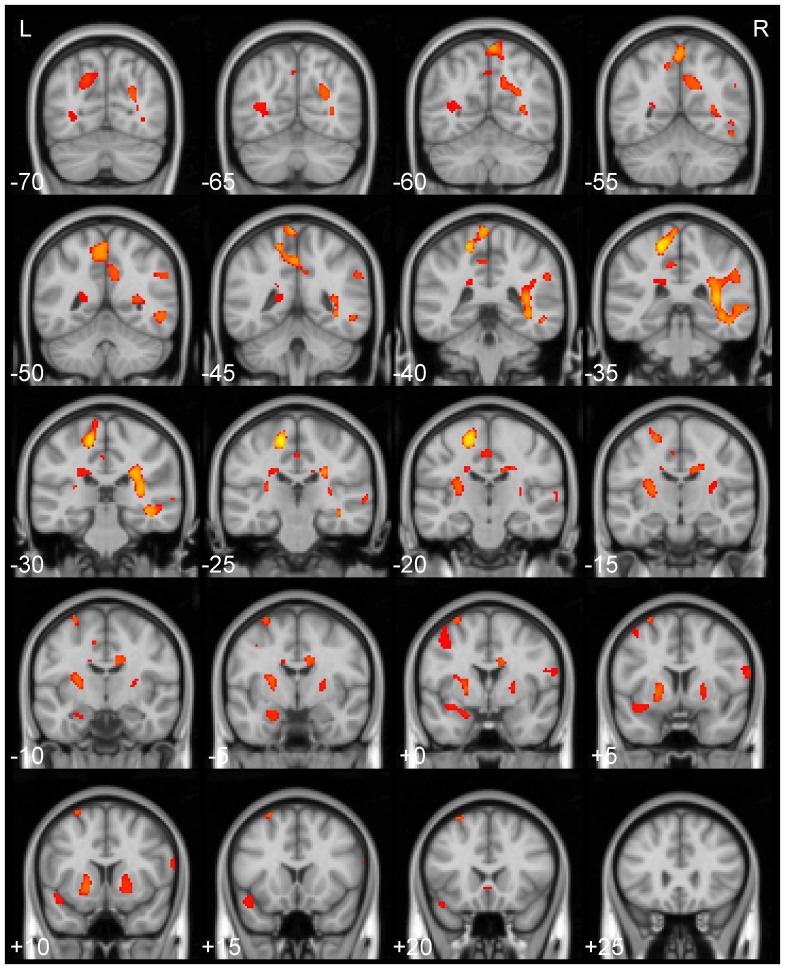
Whole-brain QSM group results. Spatial distribution of thresholded (P_TFCE_<0.05) magnetic susceptibility (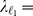
1250) differences between AD and elderly control groups overlaid onto the MNI152 template.

Spatially normalised quantitative magnetic susceptibility maps were compared between AD patients and elderly controls. In order to estimate the spatial distribution of QSM abnormalities in the whole AD brain, an inherently unbiased cluster-based analysis method was applied. Figure 8 shows the TFCE-corrected results for the QSM comparison (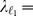
1250) of N = 8 AD versus N = 8 age-matched controls (AD values larger than those from controls). QSM alterations were found in grey and white matter tissue, specifically in the putamen bilaterally, in the left amygdala and in posterior cerebral areas. The most intense clusters were found – with relative confluence – in temporo-parietal white matter and – more scattered – in posterior parietal and occipital regions. Furthermore, widespread clusters of abnormality were also found in occipito-parietal and temporo-parietal grey- and white-matter regions. The reverse contrast did not yield any significant cluster at the present statistical threshold level.

## Discussion

This study presents a detailed methodological QSM framework for semi-automated magnetic susceptibility measurements, and offers a proof of concept of its strong potential to yield new insights in degenerative brain diseases such as Alzheimer's disease.

Dealing first with the technical aspects, it was found that the phase of the complex signal can be robustly unwrapped using a noniterative Laplacian-based method [65], [66], [69]. The RF bias-field in the magnitude image can be corrected with N4-ITK [64], and brain masks can be reliably calculated with BET2 [62] (see Fig. 1). Then, in order to remove the spurious extra-cerebral contributions from the measured phase, two previously proposed methods were tested: homodyne high-pass filtering [31] and a projection onto dipole fields (PDF) based approach [56] (see Fig. 1). As it was expected, the latter method outperformed the standard SWI filtering approach (see Fig. 3). Subsequently, the ill-posed inversion problem – of finding the magnetic susceptibility source distribution that optimally predicts the PDF-derived local reaction field – was addressed using two previously proposed regularisation approaches: one that promotes small values in the spatial gradient of the solution by applying a squared 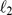-norm (sum of squares) penalty [42] (see Eq. 6); and a second regulariser that further sparsifies the gradient component by trading data fidelity with the resulting 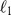-norm (sum of absolutes) of the masked gradient transform [41] (see Eq. 7). The latter appears to be, in theory, preferable because it favours highly compartmentalised solutions while still sharing sharp edges with the magnitude image [42]. This was also observed qualitatively with the present implementation (see Fig. 5), but it was noted that gradient sparsity gets promoted at the expense of anatomical detail; this will be further discussed below.

Finding the optimal regularisation parameter is key for accurate QSM [89]. In the present study, we explored the hypothesis that an optimal parameter range should not only maximise consistency with the measured data, but should also improve both serial stability and sensitivity to abnormal magnetostatic behaviour in a clinical cohort. We found that 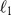-norm regularisations with *λ* = 1250 not only yielded the most faithful solutions and performed robustly in serial measurements (see Fig. 4), but also produced some of the largest group differences between AD patients and controls (see Fig. 6); all with relatively unattenuated quantitative values (see Fig. 7A). Though the pattern is clear, due to the relatively small number of subjects, it should be noted that the present optimisation might be idiosyncratic to this cohort and image acquisition.

It must also be stressed that QSM values do not represent absolute magnetic susceptibilities; this is because the coefficient at the centre of the magnetic dipole kernel in Fourier domain (see Theory S1) is unknown, and can only be approximated to satisfy boundary conditions [66]. As a result, the magnetic susceptibility contrast measured with MRI must be interpreted as a relative measure that must be normalised to a reference region. White matter tissue – in particular the splenium of the corpus callosum – has been proposed as an optimal reference region [90], but this is not adequate in the context of Alzheimer's disease where widespread white matter tract degeneration features in very early disease stages [91] and could possibly exert an influence. In order to circumvent the use of white matter as reference tissue, we calculated magnetic susceptibilities relative to that from a posterior ventricular region (see Fig. 2B). The tightly concordant results obtained from the serial experiment (see Fig. 4B and 7A) confirmed the validity of such approach. Nevertheless, future work in larger clinical datasets can systematically address the issue of whether this approach, or another reference region, is optimal to study Alzheimer's disease.

An additional observation arises from the relative nature of QSM and the various attempts that have been made to compare susceptibility values across studies [42], [92]. Though concordant overall in their regional profile, a large spread of quantitative values have been reported for asymptomatic healthy subjects; this can be attributed to age differences, choice of inversion method or parameterisation as well as variable reference selection. The quantitative values obtained in the present study were systematically lower than those reported in the literature (for review see [92]). This must be caused by the present choice of reference region (see Fig. 2B), because susceptibility differentials between structures were overall consistent with those in other studies. Our reference region was manually delineated for every subject bilaterally on the posterior end of the ventricular space, excluding partial volume contamination and large susceptibility deviations. Note that reference masks including poorly compartmentalised ventricular cerebrospinal fluid may lead to smaller reference values, which highlight the relevance of proposing standardisation strategies for normalising QSM data.

Turning to the findings of the proof of concept study, the results here suggest considerable potential for magnetic susceptibility measured with MRI – as a viable and robust postprocessed contrast mechanism – to provide novel insights in aging and neurodegenerative diseases. These results constitute, what to our knowledge is, the first attempt to estimate QSM alterations in the AD brain, with the most striking deep grey matter feature being a marked increase in magnetic susceptibility in the putamen (see Fig. 6 and 7). The caudate nucleus and the amygdala showed similar behaviours (see Fig. 7B) though differences were less statistically pronounced (see Fig. 6).

The impact of the ageing process on magnetic susceptibility was also apparent in most subcortical structures – except for the globus pallidus – and in whole grey and white matter tissue segments. Though little can be inferred from the present N = 3 young controls dataset; interestingly, these results are overall in agreement with the young-elderly sensitivity step-ups previously observed in the striatum using FDRI, SWI-phase and QSM [90], [93]–[95], which illustrate the stability of the QSM technique and its high sensitivity to changes related to the ageing process.

An important corollary from the present study is that the AD-related magnetic susceptibility alterations measured in the putamen can be observed regardless of the type of penalty or choice of regularisation parameter within a large range (see Fig. 6 and 7A). Our results agree with those from previous experiments that showed over-regularised solutions resulting in poorer susceptibility contrast [38], [39], [78]; though disagree with the conclusive statement that 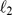-norm penalties systematically yield unreliable solutions [39], [42]. Figure 7A replicates the former observation, but we cannot conclude from the results shown in Figure 6, that Tikhonov-like regularised maps – albeit slightly noisier – are overall inferior to their 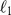-norm counterpart. Figure 5, on the contrary, suggests that there may be scenarios – when studying for example small or low susceptibility contrast structures in isolation – where the enhanced structural detail observed in certain areas of 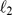-norm derived maps might be beneficial to spatially disambiguate the underlying anatomy.

Returning to the putamenal QSM abnormality, the findings were compared to hippocampal atrophy so as to gauge how significant the putamen effect might be in contrast to the most established MRI biomarker in AD [84]. This is an important issue because degeneration in AD is widespread so there is the potential to find “significant” effects that nonetheless have miniscule effect sizes compared to established markers. To this end, as shown in Figure 7A, the separation of AD from controls in the putamen suggests the statistical effect from QSM data is at least as strong as that from hippocampal atrophy (Fig. 7C).

Understanding iron metabolism in the human brain has long been an active area of research [2], [3], [6], [12], [27], [28], [96], and it has recently received increasing clinical interest because abnormally high iron concentrations have been consistently reported in a variety of neurological disorders [7]–[9], [17], [25], [97], [98]. In AD, specifically, disrupted iron homeostasis is thought to play an important role in the neurodegenerative cascade [14]–[18], [99], though its precise mechanisms are not yet fully elucidated.

It is beyond the realms of the QSM technique to explain the present AD results uniquely in terms of the molecular biology of iron, but some of its more relevant aspects will be briefly contextualised here. It is thought that iron overload may trigger excess concentration of reactive radical species; this leads to oxidative damage, which deleteriously impacts the neural system [4], [7], [9], [10]. A link has also been found between β-amyloid proliferation and increased neurotoxicity in the presence of iron [23], [24]. In addition, it has been suggested that ferritin in AD may contain increased iron concentration [100], while it has been shown that iron-rich deep grey matter structures do not appear to saturate the susceptibility-weighted MRI signal [101]. Ferritin is found in abnormally high concentrations in the AD basal ganglia, offering a precedent to the present results using QSM. Furthermore, recent evidence from the Dominantly Inherited Alzheimer Network (DIAN) suggests that the basal ganglia are the earliest and most intense accumulators of β-amyloid in those genetically predisposed to develop AD in the future [102]. Previous *in vivo* MRI experiments also detected abnormalities in the AD basal ganglia [17], [98], [103], confirming that the present methodology represents a viable and accurate alternative to SWI, FDRI and transverse relaxation rate measurements to study iron deposition in neurodegenerative diseases such as AD.

Iron is the most abundant transition metal in the human brain [2], and therefore a major source of paramagnetism in grey and white matter structures [26]–[28]. But the mineralisation of the basal ganglia in degenerative diseases encompasses a myriad of potential chemical perturbations, some of which – in addition to iron – involve species that may also respond to an applied magnetic field [15], [100], [104]–[106]. Magnesium and calcium, for example, are paramagnetic in their elemental state due to the effect of conduction electrons, but they are typically diamagnetic when they lose their delocalised electrons to form compounds. Copper and zinc are metals with filled electron orbitals, which result in slightly diamagnetic moments similar to those from soft tissue and water. Copper atoms, however, can lose two electrons to form a Cu^2+^ ion, resulting in Cu(II) compounds that are typically paramagnetic. The aluminium atom is also paramagnetic – it has three valence electrons, one of which is unpaired in the outermost shell; though the aluminium ion, Al^3+^, has no valence electrons at all. The effect of the abnormal expression of such metallic species on magnetic susceptibility measurements might be subtle overall, but they are known to be constituents of many brain structures [104], [106], [107]. It is therefore at least conceivable that their effect might not be negligible in the susceptibility-weighted MR signal. Moreover, it has been found that transverse relaxation rates in the basal ganglia not only predict iron deposition as a function of age, but also account for an additional regional cofactor that must be driven by other trace elements [108]. The results from these studies suggest that caution should be exercised when interpreting magnetic susceptibility differences uniquely as iron-related changes, particularly in disease, because current QSM approaches are unable to characterise the exact chemical configuration underlying abnormal magnetostatic behaviours. And although postmortem studies have shown that iron overload is the most likely candidate for driving such alterations [26]–[28], at present, disentangling whether the magnetic susceptibility differences measured by QSM are predominantly driven by changes in iron concentration, its valence or both, is not possible. Besides, the overall effect from other magnetically responsive ions/molecules – that might be involved in a variety of neuropathological processes – has not yet been confirmed to be negligible; hence it will not be ruled out here.

A unique feature of QSM is its ability to produce volumetric quantitative susceptibility maps in short scanning times. The voxel-based results shown in Figure 8 represent the first whole brain assessment of this kind in AD and, again, highlight the magnetic susceptibility alterations identified in the striatum by the regional analysis. The remaining statistical effects identified beyond deep grey matter need to be interpreted with a little caution until replicated. The group sizes are small; also it is unclear whether the inherit problem of misregistration in two-population voxel-based analyses [109] could generate artefacts with this new data type. Nevertheless, it is reassuring that: the whole-brain analysis could identify the striatal lesion that was demonstrated by regional data extraction; that the reverse contrast (controls greater than AD) was completely negative; and that certain blobs appeared to be following anatomical boundaries (see white matter blob at y = −35 mm in Fig. 8); they all argue that the results are not spurious. It is interesting to note that the most extensive and significant changes were found in posterior temporo-parietal white matter (see Fig. 8). The white matter in this region has the greatest predilection for lobar haemorrhage and microbleeds leading to the possibility that QSM may be detecting signals related to amyloid angiopathy [110], [111]. Such speculation, of course, will need confirmation in future clinical studies. It is also important to stress that QSM behaviour is still poorly understood in predominantly uniaxial microstructural environments such as white matter; this is due to the inherent rotational variance of the magnetic susceptibility MRI measurement with respect to the orientation of the main field [66], [112]–[116]. The reconstruction of a susceptibility tensor has been proposed [117], which might hold potential – aided by diffusion tensor information [118] – to explain whether QSM abnormalities in diseased white matter are primarily driven by axonal loss, myelin-iron imbalance, angiopathy or other factors.

In contrast to white matter, little convincing evidence was found for changes in the cortical ribbon though this, too, should be explored further in larger studies. Normal inter-subject variability in the topography of the cortex may well mean that the pilot study was underpowered to detect changes; for instance, although cortical atrophy is a known feature of AD, an N = 8 subject/group grey matter density contrast would also typically show no abnormalities in a voxel-based analysis.

An additional contributor to systematic magnetic susceptibility change in neurodegenerative disease could be differential venous-blood oxygen saturation levels in large vessels [58], [114], [119]. Although such effects might still play a role in QSM measurements, and needs to be further investigated, pilot data suggests that such contribution might be negligible relative to those from other sources [120].

A final caveat to the pilot study is that the specificity of the observed QSM abnormalities to AD will need to be confirmed by studying other neurodegenerative conditions. Noting in the present study that there were changes in the putamen between young and old controls, it may be that putamenal alterations in AD represent an exacerbated ageing-like process, and may in turn be common to many neurodegenerative diseases [121].

In conclusion, the present study demonstrated the potential of QSM as an MRI biomarker. The exact origins of magnetic susceptibility alterations require further investigation, but this work shows that QSM is ready for larger-scale clinical studies; it has the potential to provide unique etiological and diagnostic information about tissue compositional changes in neurodegenerative diseases as well as in the ageing brain. With the advent of faster [69] and more advanced regularisation strategies [43], [122], and combining multi-contrast information [118], [123]–[125], the applicability of magnetic susceptibility mapping to study the human brain is only bound to grow.

Code for QSM reconstruction can be requested from the corresponding author.

## Supporting Information

Introduction S1 **Magnetic susceptibility in the human brain.** Introduction to magnetic susceptibility and its relevance to study the role of iron (and other paramagnetic substances) in neurodegenerative diseases using MRI.
(PDF)

Theory S1 **A primer on QSM background theory.** Brief theoretical description of the Fourier-based approximation relating magnetic susceptibility sources with nonlocal field inductions.
(PDF)
