# Supplementary material for: In Vivo Quantitative Susceptibility Mapping (QSM) in Alzheimer's Disease
Source: PLoS One. 2013 Nov 21;8(11):e81093. doi: 10.1371/journal.pone.0081093 (PMC3836742; doi:10.1371/journal.pone.0081093)
Supplement: Introduction S1 — Magnetic susceptibility in the human brain. Introduction to magnetic susceptibility and its relevance to study the role of iron (and other paramagnetic substances) in neurodegenerative diseases using MRI. (PDF) [file pone.0081093.s001.pdf]

"*In vivo* **quantitative susceptibility mapping (QSM) in Alzheimer's disease**"
Acosta-Cabronero J *et al*. Plos One

SUPPORTING INFORMATION – INTRODUCTION S1

Magnetic susceptibility in the human brain

In a magnetised molecular ensemble *e.g.* in a biomaterial of interest placed at the isocentre of an MRI magnet, the sum of all its nuclear moments can be synthesised to a net magnetisation vector field, $\mathbf{M}$, measured in amperes per metre (A/m); which is related to the applied magnetic field, $\mathbf{H_0}$ – also a vector field measured in A/m – as:

$$\mathbf{M} = \chi \mathbf{H_0} \qquad \text{(Eq. 1)}$$

The proportionality constant, χ, is the volume magnetic susceptibility – a dimensionless physical quantity, typically of the order of parts per million (ppm), that describes the degree of magnetisation that a static magnetising force induces in a substance. Magnetic susceptibility enables the classification of materials as superconducting ($\chi \approx -1$), diamagnetic ($-1 < \chi < 0$), paramagnetic ($\chi > 0$) or ferro-/superparamagnetic ($\chi \gg 0$).

In this context, χ is a bulk measure and as such, it is a complicated function that depends on the exact molecular arrangement and local temperature. More specifically, magnetic susceptibility is driven by the complex interaction between the effects that the external field imposes: (i) on electronic orbital motions, (ii) on the alignment of nuclear and electronic magnetic moments, and for certain metallic environments, (iii) on the motion of conduction electrons [1]. It also accounts for the effect of inherent thermal fluctuations if the time-scale of the measurements is not infinitesimally short, as is the case for MRI.

It is beyond the scope of the present manuscript to provide a full description of all field-induced mechanisms that contribute to magnetic susceptibility – for

review see [2]; however, two fundamental magnetic phenomena, not only in biological tissue but in any medium, represent the cornerstone of magnetic susceptibility in the human brain and will be briefly introduced here: (i) the systematic adoption of a lowest-energy configuration ensures predominant alignment of the nuclear magnetic moments along the orientation of the applied field. In other words, nuclei with nonzero dipole moments – *i.e.* with even atomic numbers – always have a positive contribution to the net susceptibility, whereas (ii) alterations in electron orbital motion – also by direct interaction with the external field – yield magnetic moments that are always opposed to the main field ($\chi < 0$) [3]. Though (i) and (ii) are competing effects, the electron contribution to magnetic susceptibility is always stronger than that for nuclei, which explains why most substances in nature tend to be slightly diamagnetic. Soft tissue and water are two such examples but there are some exceptions: for transition metals or rare earth elements with unpaired electron orbitals such as iron, manganese or gadolinium, the para-, ferro- or superparamagnetic contribution due to the preferential alignment of their electronic dipole moment with the main field is much stronger and overrides the slight diamagnetism induced by orbital alterations. In addition, other nonferromagnetic metallic elements – including alkaline earth metals such as magnesium or calcium – possess delocalised conduction electrons with magnetic moments that preferentially align with the external field, which also give rise to a paramagnetic susceptibility component that counteracts the predominant diamagnetism.

The prevalence of such elements in the human brain is low relative to the total volume of diamagnetic soft tissue, water and lipids; but in specific regions such as the corpus striatum (caudate nucleus, putamen and globus pallidus) and in the mesencephalon (substantia nigra and red nucleus), iron atoms are present in large concentrations as they are sequestered and stored by the metalloprotein ferritin [4]. In these areas, they can be found forming a ferric oxyhydroxide-phosphate core surrounded by a spherical protein shell known as apoferritin [5].

Ferritin is involved in iron detoxification both intra- and extra-cellularly, and its uptake mechanism is thought to involve the oxidation of Fe(II) and the

incorporation of Fe(III) into a ferric oxyhydroxide lattice [6,7]. Storing iron in a soluble, nontoxic form – *i.e.* in ferritin cores – is essential to maintain brain iron homeostasis; this is because ferrous ions, which are demanded by cells, are highly reactive in the extracellular space and may therefore stimulate the overexpression of free peroxide radicals – which are in turn linked to neuronal death [8]. Brain iron, however, is present in many other forms: for example, ferric ions also bind to the weakly-magnetic glycoprotein transferrin for transportation in plasma [9,10]; a breakdown product of intra-cellular ferritin known as haemosiderin is present in haemorrhages [11]; and it can be found in large amounts in red blood cells as haemoglobin. Interestingly, oxygen-carrying arterial haemoglobin is not highly paramagnetic chiefly due to the cancellation effect produced by the single unpaired electron of the superoxide ion (O_2^-), which aligns antiferromagnetically with that from low-spin ferric ions [12]. It is only after oxygen has transferred to tissue that the magnetic susceptibility of venous blood (deoxyhaemoglobin) is dominated by the presence of iron [13,14]; this, in turn, is the basis for the blood-oxygen-level-dependent (BOLD) signal measured in functional MRI.

REFERENCES

1. Van Vleck JH (1932) The theory of electric and magnetic susceptibilities. London: Oxford University press. pp. 1-26.
2. Schenck JF (1996) The role of magnetic susceptibility in magnetic resonance imaging: MRI magnetic compatibility of the first and second kinds. Med Phys 23: 815-850.
3. Van Vleck JH (1932) The theory of electric and magnetic susceptibilities. London: Oxford University press. pp. 89-94.
4. Hallgren B, Sourander P (1958) The effect of age on the non-haemin iron in the human brain. J Neurochem 3: 41-51.
5. Harrison PM, Fischbach FA, Hoy TG, Haggis GH (1967) Ferric oxyhydroxide core of ferritin. Nature 216: 1188-1190.

6. Hoy TG, Harrison PM, Shabbir M (1974) Uptake and release of ferritin iron. Surface effects and exchange within the crystalline core. Biochem J 139: 603-607.

7. Chasteen ND, Harrison PM (1999) Mineralization in ferritin: an efficient means of iron storage. J Struct Biol 126: 182-194.

8. Smith MA, Harris PL, Sayre LM, Perry G (1997) Iron accumulation in Alzheimer disease is a source of redox-generated free radicals. Proc Natl Acad Sci U S A 94: 9866-9868.

9. Crichton RR, Charloteaux-Wauters M (1987) Iron transport and storage. Eur J Biochem 164: 485-506.

10. Connor JR, Menzies SL, St Martin SM, Mufson EJ (1990) Cellular distribution of transferrin, ferritin, and iron in normal and aged human brains. J Neurosci Res 27: 595-611.

11. Andrews SC, Treffry A, Harrison PM (1987) Siderosomal ferritin. The missing link between ferritin and haemosiderin? Biochem J 245: 439-446.

12. Chen H, Ikeda-Saito M, Shaik S (2008) Nature of the Fe-O2 bonding in oxy-myoglobin: effect of the protein. J Am Chem Soc 130: 14778-14790.

13. Ogawa S, Lee TM (1990) Magnetic resonance imaging of blood vessels at high fields: in vivo and in vitro measurements and image simulation. Magn Reson Med 16: 9-18.

14. Petridou N, Wharton SJ, Lotfipour A, Gowland P, Bowtell R (2010) Investigating the effect of blood susceptibility on phase contrast in the human brain. Neuroimage 50: 491-498.
