# Supplementary material for: In Vivo Quantitative Susceptibility Mapping (QSM) in Alzheimer's Disease
Source: PLoS One. 2013 Nov 21;8(11):e81093. doi: 10.1371/journal.pone.0081093 (PMC3836742; doi:10.1371/journal.pone.0081093)
Supplement: Theory S1 — A primer on QSM background theory. Brief theoretical description of the Fourier-based approximation relating magnetic susceptibility sources with nonlocal field inductions. (PDF) [file pone.0081093.s002.pdf]

"***In vivo* quantitative susceptibility mapping (QSM) in Alzheimer's disease**"

Acosta-Cabronero J *et al*. Plos One

SUPPORTING INFORMATION – THEORY S1

A primer on QSM background theory

Field perturbation

When placing a medium with nonzero magnetic susceptibility in a static magnetic field, local magnetisations perturb the previously homogeneous field by inducing a reaction field that is no longer local. The perturbed magnetic field, $\mathbf{H}$, can be expressed as a composite function of three superposed fields: (i) the applied magnetic field, $\mathbf{H_0}$, assumed here to be constant everywhere; (ii) the locally-sourced self-demagnetising reaction field, $\mathbf{H^L}$, from the interaction of all magnetic moments within the brain; and (iii) the shimming residual, $\mathbf{H^E}$, *i.e.* a demagnetising field component originating from outside the brain by fat, bone and paramagnetic oxygen contained in air-filled cranial cavities. This can be formulated as follows [1,2]:

$$\mathbf{H} = \mathbf{H_0} + \mathbf{H^L} + \mathbf{H^E} \qquad \text{(Eq. 1)}$$

The contribution of the demagnetising fields, which are proportional to $\mathbf{H_0}$ but several orders of magnitude weaker, is only relevant to the measurements along the direction of the main magnetic field; this orientation, by convention, defines $\mathcal{Z}$-axis. The vector fields, $\mathbf{H^E}$ and $\mathbf{H^L}$, can thus be simplified to scalar fields representing their $\mathcal{z}$-component *i.e.* $\mathrm{H}_{\mathcal{z}}^{\mathrm{E}}$ and $\mathrm{H}_{\mathcal{z}}^{\mathrm{L}}$.

Field induction

Magnetic fields are not directly measurable, it is the macroscopic magnetic induction, **B** – also a measure of field strength (in tesla, T) characterised by the

density of field lines (or magnetic-flux density) – that MRI approaches are directly sensitive to:

$$\begin{aligned}\mathbf{B}(\mathbf{r}) &= \mu_0[\mathbf{H}(\mathbf{r}) + \mathbf{M}(\mathbf{r})] \\ &= \mu_0[1 + \chi(\mathbf{r})] \cdot \mathbf{H}(\mathbf{r})\end{aligned} \qquad \text{(Eq. 2)}$$

In Eq. 2, μ_0 ($= 4\pi \cdot 10^{-7}$ henries per metre) is the magnetic permeability in a vacuum. Therefore, $\mathbf{B_0} = \mu_0 \mathbf{H_0}$ is the magnetic field induced by $\mathbf{H_0}$ in "free space".

"Sphere of Lorentz" correction

^{1}H-MRI signal phases reflect the effective microscopic magnetic induction experienced by hydrogen nuclei; this, however, is **B'** – the Lorentz "cavity field" – not **B**. **B'** corrects for the cancellation effect due to the interaction between the magnetic moment of the hydrogen nucleus with those from other nuclei and electronic shells in the vicinity [3]:

$$\mathbf{B}'(\mathbf{r}) = \left[1 - \tfrac{2}{3}\chi(\mathbf{r})\right] \cdot \mathbf{B}(\mathbf{r}) \qquad \text{(Eq. 3)}$$

neglecting chemical shift effects.

Combining then Eq 1, 2 and 3, while keeping on a first-order approximation, the magnetic field induction experienced by hydrogen protons along $\mathcal{Z}$, $\mathrm{B}'_{\mathcal{Z}}$, can be defined as [1,2]:

$$\mathrm{B}'_{\mathcal{Z}}(\mathbf{r}) = \frac{\mu_0 H_0}{3}\chi(\mathbf{r}) + \mu_0[H_0 + \mathrm{H}^{\mathrm{E}}_{\mathcal{Z}}(\mathbf{r}) + \mathrm{H}^{\mathrm{L}}_{\mathcal{Z}}(\mathbf{r})] \qquad \text{(Eq. 4)}$$

The magnetic dipole kernel

Salomir *et al.* derived a theoretical approximation for the local reaction field, $\mathrm{H}^{\mathrm{L}}_{\mathcal{Z}}$ [2]; this expression can be substituted into the right-hand side term of Eq. 4,

which equates to the additive scalar field of magnetic flux densities along $\mathcal{Z}$. The local self-demagnetising field induction, $\mathrm{B}_z^{\mathrm{L}}$, can therefore be expressed as:

$$\mathrm{B}_z^{\mathrm{L}}(\mathbf{r}) = B_0 \mathcal{D} \chi(\mathbf{r}) \qquad \text{(Eq. 5)}$$

where,

$$\mathcal{D} = \mathcal{F}^{-1} \mathrm{D}(\mathbf{k}) \cdot \mathcal{F} \qquad \text{(Eq. 6)}$$

with,

$$\mathrm{D}(\mathbf{k}) = \frac{1}{3} - \mathrm{K_d}(\mathbf{k}) \qquad \text{(Eq. 7)}$$

and,

$$\mathrm{K_d}(\mathbf{k}) = [|\mathbf{k}|^{-1} \cdot \mathrm{k_z}(\mathbf{k})]^2 = \frac{\mathrm{k_z}^2(\mathbf{k})}{\mathrm{k_x}^2(\mathbf{k}) + \mathrm{k_y}^2(\mathbf{k}) + \mathrm{k_z}^2(\mathbf{k})} \qquad \text{(Eq. 8)}$$

The wave-vector $\mathbf{k} = \left[\, \mathrm{k_x}(\mathbf{k}),\ \mathrm{k_y}(\mathbf{k}),\ \mathrm{k_z}(\mathbf{k}) \,\right]$ is the Fourier conjugate of **r,** $\mathrm{K_d}$ represents k-space scaling coefficients, D defines the unit magnetic dipole field response in Fourier domain, and $\mathcal{D}$ is the related spatial operator that readily enables source-to-field induction transformations. Eq. 5 – equally obtained on different theoretical grounds [2,4,5] – provides a fast Fourier-based approximation to the magnetostatic boundary problem of inferring the local magnetic induction generated by an arbitrary-shaped medium. The scaling coefficient at the centre of the reciprocal space ($k_{\mathrm{x,y,z}} = 0$), however, is undetermined; hence to satisfy appropriate boundary conditions, an approximate solution has been derived, $\mathrm{K_d}(\mathbf{k_0}) = -2/3$ [6].

REFERENCES

1. Li L (2001) Magnetic susceptibility quantification for arbitrarily shaped objects in inhomogeneous fields. Magn Reson Med 46: 907-916.

2. Salomir R, de Senneville BD, Moonen CTW (2003) A fast calculation method for magnetic field inhomogeneity due to an arbitrary distribution of bulk susceptibility. Concepts Magn Reson Part B Magn Reson Eng 19B: 26-34.
3. Lorentz HA (1916) The theory of electrons and its applications to the phenomena of light and radiant heat: a course of lectures delivered in Columbia University, New York, in March and April 1906. 2nd ed. Leipzig: B.G. Teubner. pp. 132-139.
4. Marques JP, Bowtell R (2005) Application of a Fourier-based method for rapid calculation of field inhomogeneity due to spatial variation of magnetic susceptibility. Concepts Magn Reson Part B Magn Reson Eng 25B: 65-78.
5. Koch KM, Papademetris X, Rothman DL, de Graaf RA (2006) Rapid calculations of susceptibility-induced magnetostatic field perturbations for in vivo magnetic resonance. Phys Med Biol 51: 6381-6402.
6. Li W, Wu B, Liu C (2011) Quantitative susceptibility mapping of human brain reflects spatial variation in tissue composition. Neuroimage 55: 1645-1656.
